# Editorial: Genome editing strategies for augmenting crop resilience against climate change

**DOI:** 10.3389/fgeed.2026.1813338

**Published:** 2026-03-13

**Authors:** Wadzani Palnam Dauda, Amolkumar U. Solanke, Ramesh Namdeo Pudake

**Affiliations:** 1 Faculty of Agriculture, Federal University Gashua, Yobe, Nigeria; 2 Indian Council of Agricultural Research -National Institute for Plant Biotechnology, New Delhi, India; 3 Amity Institute of Nanotechnology, Amity University Uttar Pradesh, Noida, India

**Keywords:** crop protection, crop engineering, genome breeding, genome edited (GenEd), genome edited crops, abiotic stress

## 1 Introduction

Global stability and increase of crop yield has been affected negatively in recent times due to the frequent incidents of temperature and rainfall, variations leading to heat, cold, drought and salinity stress (Abdulwahid et al., 2026). These stressors occur as shifts in pest and disease development, ion toxicity, canopy temperature, water balance, oxidative damage and several other forms. Harnessing the precision of genome editing tools like CRISPR-Cas system and other advanced tools can be used to incorporate defined alleles in elite backgrounds, which can then be evaluated in controlled and field condition, reducing the long-time frame usually required for traditional introgression programs (Azameti and Dauda, 2021; Kalaitzandonakes et al., 2023). There is a pressing need to translate cutting-edge biotechnological advancements into actionable solutions, ensuring the timely development of climate-resilient crops. This Research Topic was framed around translational genomics and requested research work that links stress-gene identification to actionable edits. Its intended scope includes the evaluation of genome editing platforms, optimization of protocols and delivery, and validation under conditions aligned with climate risk. The Research Topic also explicitly includes environmental and regulatory considerations and emphasizes collaborative approaches and field trials that test edited lines in production settings.

## 2 Highlights of the contributions

### 2.1 Targets and edit types for stress response and adaptation

In the review article, Chavhan et al. provided a comprehensive synthesis on the application of gene editing for climate-resilient crops using CRISPR/Cas9, base editing, and prime editing. It elaborates when targeted mutagenesis, direct base conversion without double-strand breaks, or templated edits are suited to trait development. They discuss stress-response regulators and ion or osmotic homeostasis components, including DREB, HSP, SOS, ERECTA, HsfA1, and NHX. They connect these targets to trait domains used in crop improvement, including root development, water use efficiency, photosynthesis, membrane stability, ion homeostasis, osmotic adjustment, and oxidative stress response. The review also identifies constraints that condition translational success includes off-target effects, delivery limitations, and regulatory barriers.

### 2.2 Product design steps that reduce regulatory and breeding friction

Rafi et al. address selectable marker genes, which are useful during transformation but can complicate biosafety assessment and acceptance when retained in final events. Using tobacco, they re-transformed established transgenic lines with a multiplex CRISPR/Cas9 vector encoding four guide RNAs that target sequences flanking a DsRED marker cassette. They report loss of red fluorescence in about 20% of regenerated shoots and confirm marker cassette excision in a subset, indicating an excision efficiency around 10%. They further confirm the absence of DsRED expression in marker-deleted lines while maintaining expression of Cas9 and the gene of interest, and report recovery of Cas9-free, marker-free plants through segregation in the T1 generation. In climate-resilience pipelines that still use transgenic steps for delivery, such marker excision strategies support event stewardship, trait stacking, and integration into breeding programs.

### 2.3 Editing structural variation and gene dosage for stress-linked traits

Park et al. explored the novel concept on copy number variation (CNV), a structural variant class that is increasingly documented in crop pan-genome resources but remains difficult to test experimentally because duplicated gene blocks can share high sequence identity. In rice, they apply a Cas9 strategy that uses cytosine extensions to conventional single-guide RNAs to generate frameshift mutations across copies of OsGA20ox1 and thereby modulate functional gene copy number. They report that OsGA20ox1 copy number is a determinant of seedling vigor in their CNV-modified materials, and they also use Cas3, a nuclease suited to large deletions, to decrease copy number of OsMTD1. They couple genome editing with verification approaches that include droplet digital PCR, Sanger sequencing, and bioinformatics analyses, illustrating an experimental route to isolate CNV effects without changing genetic background.

### 2.4 Governance and capacity as determinants of deployment

Adegbaju et al. did a synthesis on the enabling conditions that shape whether genetic engineering and genome editing can contribute to food security, using Nigeria as a case study. They describe national policy approaches aimed at capacity building, research and development, and commercialization of biotechnology. They also highlight a trust barrier relevant to deployment and uptake when transgenic crops are introduced mainly for confined field trials after being produced outside the country, and they link this to public suspicion and the framing of biotechnology as external technology. The review also documents policy movement on genome editing regulation in Africa, noting that Nigeria amended biosafety legislation to include genome-edited products and that resulting guidelines have been adopted since 2020, alongside uptake of related approaches in other African countries. For climate-resilient crop delivery, these governance features are not an external add-on: they influence which product types are pursued, how evidence is generated, and how edited varieties are advanced within national seed systems.

## 3 Emerging insights and research directions

Priorities that follow from these contributions are operational and measurable. In addition, best practice verification frameworks now emphasize the use of targeted sequencing and quantitative assays to confirm the molecular state of edited loci, including long-read sequence context to detect structural variants and detailed copy number variation assessment (Tsakirpaloglou et al., 2023) provide a foundation for this approach. Where feasible, delivery systems that avoid stable integration, such as Cas9 ribonucleoprotein or protoplast-mediated editing, should be prioritized to facilitate marker excision and simplify downstream segregation planning.

### 3.1 Closing perspective

The manuscripts in this Research Topic outline a translation chain that links target discovery and editing modality selection to event design, molecular verification, and deployment conditions. A research message across the set is that climate adaptation traits often require intervention at regulators, transport and homeostasis systems, and genomic features such as CNV, which makes experimental design and phenotyping strategy central to interpretation. An enabling deployment message is that product design decisions, including marker retention or removal and the choice of regulatory pathway for genome-edited products, influence speed, stewardship, and regional uptake. These lessons align with the Research Topic’s translational remit by connecting the “how” of editing to the “how” of bringing edited lines into breeding and agricultural practice under climate stress.
